# Myocarditis Outbreak among Adults, Illinois, 2003

**DOI:** 10.3201/eid1110.041152

**Published:** 2005-10

**Authors:** Gregory D. Huhn, Cindy Gross, David Schnurr, Chris Preas, Shigeo Yagi, Sarah Reagan, Chris Paddock, Douglas Passaro, Mark S. Dworkin

**Affiliations:** * Centers for Disease Control and Prevention, Atlanta, Georgia, USA; † Illinois Department of Public Health, Chicago, Illinois, USA; ‡ Kane County Health Department, Aurora, Illinois, USA; § California Department of Health Services, Richmond, California, USA; ¶ University of Illinois, Chicago, School of Public Health, Chicago, Illinois, USA

**Keywords:** myocarditis, cardiomyopathy, viral, outbreak, biopsy, polymerase chain reaction, surveillance, enzyme immunoassay, immunohistochemistry, Illinois, dispatch

## Abstract

An outbreak of myocarditis occurred among adults in Illinois in 2003. Diagnostic testing of myocardial tissues from 3 patients and comprehensive tests for enterovirus and adenovirus of other specimens from patients were inconclusive. Appropriate specimen collection from patients with idiopathic cardiomyopathy and further enhancement of diagnostic techniques are needed.

Acute myocarditis is characterized by inflammatory infiltrates of the myocardium. Disease has been attributed to multiple infectious and noninfectious causes, but viruses, particularly the enteroviruses group B coxsackievirus and echoviruses, are believed to be the most common agents of infection in the United States (*1*). An infectious cause of myocarditis is usually suspected when unexplained heart failure or arrhythmia occurs in a person with a systemic febrile illness or upper respiratory tract infection. Acute myocarditis is typically sporadic, although clusters have been reported during outbreaks of viral disease (*2**,**3*). Most cases are idiopathic without a known cause (*1*). Myocardial biopsy specimens used for pathologic examination, the conventional standard for diagnosis (*4**,**5*), have been considered difficult to collect in nonfatal cases. Viruses are infrequently cultured from tissue specimens, although viral nucleic acid identification by polymerase chain reaction (PCR) assays on myocardium has recently enhanced viral detection (*6**–**8*). Viral serologic tests and PCR assays of blood, stool, urine, and nasopharyngeal specimens are adjunctive techniques for diagnosing myocarditis that have not been validated.

On March 21, 2003, the Kane County Health Department was notified about 6 cases of presumptive myocarditis and 1 case of pericarditis that occurred in patients hospitalized in Kane County, Illinois, within a 2-week period from February 26 to March 10. Five case-patients were <50 years of age, 1 of whom died within 24 hours of hospitalization. Five of the 6 case-patients were hospitalized at hospital A. Illinois Department of Public Health (IDPH) and Kane County Health Department initiated an investigation to identify additional cases and determine the cause of illness.

## The Study

On March 22, IDPH distributed a notice describing the cluster of myocarditis cases to local health departments and healthcare providers in Illinois and requested urgent reporting of similar cases. At hospital A, where most of the initial cases were diagnosed, active surveillance was instituted for patients with a clinical syndrome consistent with myocarditis or pericarditis or an upper respiratory tract illness with profound fatigue or disproportionate shortness of breath of >2 weeks' duration. For patients with suspected cases, a testing protocol was implemented, which included a 2-dimensional echocardiogram; electrocardiogram; chest radiograph; measure of serum cardiac enzymes; complete blood count; nasopharyngeal, stool, and urine samples for enterovirus assays; and acute- and convalescent-phase serologic testing for enterovirus.

A review of all records for patients with discharge diagnoses of myocarditis or cardiomyopathy at all 5 hospitals in Kane County from October 1, 2002, through March 31, 2003, was conducted to find unreported cases of myocarditis. Persons with ischemic, alcoholic, postpartum, or chronic cardiomyopathy were excluded. To determine the background number of myocarditis cases for all patients <50 years of age in Kane County, a database search of medical records during the preceding 2-year period (October 1, 2000 to September 30, 2002) at all 5 hospitals was performed by principal International Classification of Diseases, 9th revision (ICD-9) discharge diagnosis codes (Appendix).

A case of myocarditis was defined as 1) a person with myocarditis diagnosed by electrocardiogram, echocardiogram, or cardiac catheterization, which indicates the presence of unexplained arrhythmia or decreased ejection fraction without apparent cause or 2) myocardial inflammatory infiltrates on tissue pathologic examination by using the Dallas criteria (*9*) or 3) viral isolation or nucleic acid identification in myocardial tissue specimens in persons living in northern Illinois from October 1, 2002, through May 30, 2003.

Medical records of patients were reviewed, and physicians who treated case-patients were interviewed when available. Information was collected about patient demographics; antecedent illness; underlying medical condition; exposure to toxins, pets, or ill persons; recent travel; and smallpox vaccination history.

The results of echocardiograms and routine specialized laboratory tests, including enterovirus complement-fixation serologic screening, conducted by physicians who evaluated patients at hospitals, were recorded. Nasopharyngeal, urine, and stool specimens from patients were cultured for enterovirus at the IDPH laboratory. Any available serum and myocardial tissue specimens from patients were tested at the California Department of Health Services Viral and Rickettsial Disease Laboratory by using real-time PCR nucleic acid amplification (Amersham Eclipse, Piscataway, NJ, USA) and immunoglobulin M (IgM) enzyme immunoassay for detecting enterovirus and adenovirus (*10**,**11*).

Pathology reports on autopsy specimens from patients with fatal cases and myocardial biopsy specimens from patients with nonfatal cases were reviewed. Formalin-fixed, paraffin-embedded tissue from the autopsy of 1 available patients was submitted to the Centers for Disease Control and Prevention (CDC) Unexplained Deaths and Critical Illnesses (UNEX) Laboratory for Gram and calcium staining, enteroviral 5´ noncoding region gene PCR assay, and immunohistochemical staining to detect enterovirus, cytomegalovirus, influenza A, influenza B, and hantavirus.

**Figure 1:**
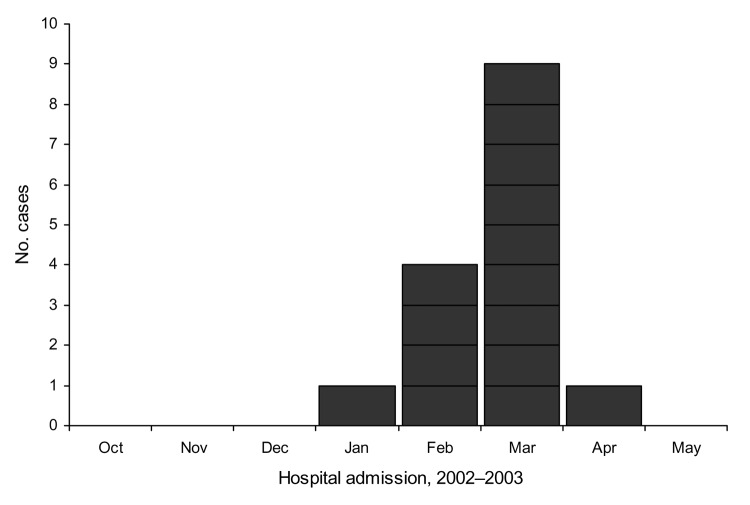
Reported myocarditis case-patients by month of hospital admission, northern Illinois, 2003. (N = 15 because the exact date of admission to hospital was unknown for 1 patient).

Sixteen cases, 1 of which (that of patient 8) was recognized through retrospective medical record review, were identified. All patients were hospitalized and admitted between January 28 through April 7 (Figure 1), and 13 patients (81%) were adults <50 years of age. Six (38%) of the 16 patients were hospitalized at hospital A during January through March. For comparison, the number of diagnoses of myocarditis in patients <50 years of age (16 patients) from October 1, 2000, to September 30, 2002, was <1 per month.

**Figure 2:**
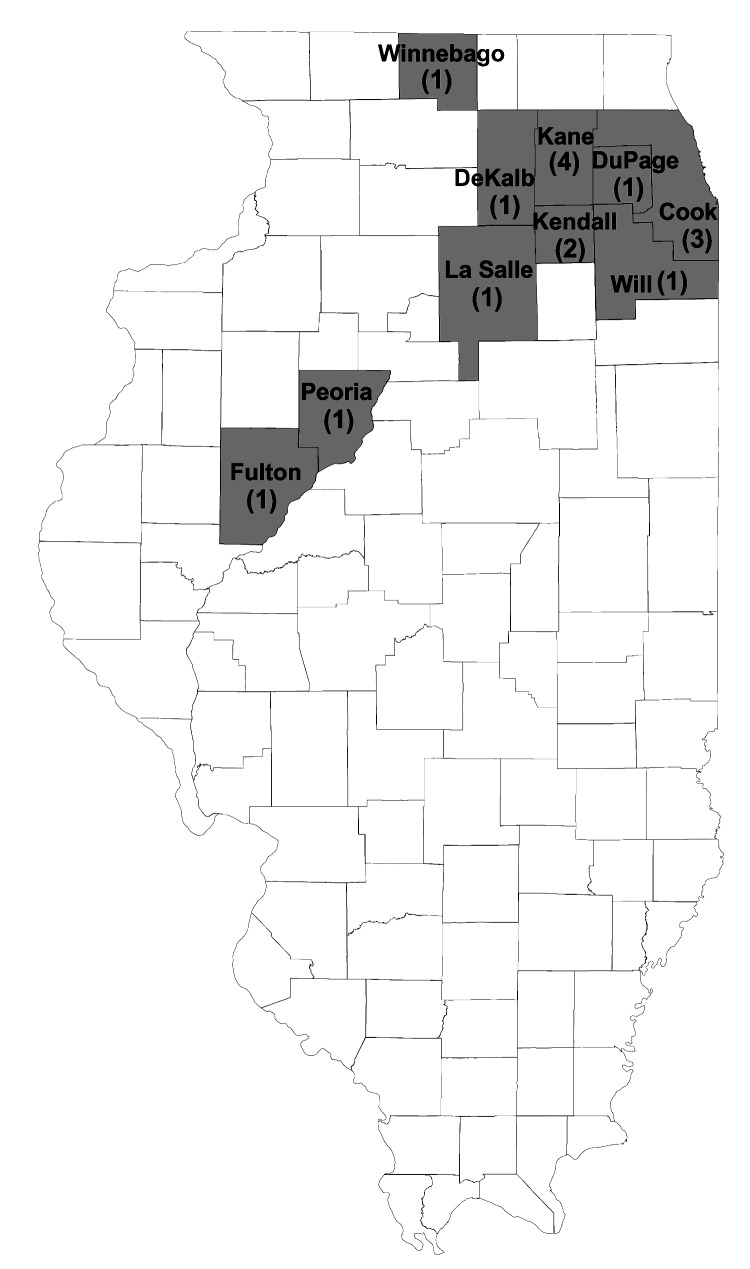
County of residence of reported myocarditis case-patients (N = 16), northern Illinois, 2003.

The median age for patients was 38 years (range 20–70 years). Among the 16 case-patients, 4 (25%) were residents of Kane County, 8 (50%) were from 5 counties bordering Kane County, and 4 (25%) were from 4 other counties in northern Illinois (Figure 2).

Thirteen case-patients (81%) had an acute, viral-like illness within 1 month before onset of myocarditis. Two female patients, 26 and 39 years of age, had ventricular fibrillation that required an automatic implantable cardioverter defibrillator (AICD) and recovered. There were 2 deaths (Table 1).

**Table 1:** Demographic and clinical features of reported myocarditis patients, northern Illinois, 2003

				Cardiac test results			
Patient and county of residence	Age/Sex	Date of hospital admission	Illness prodrome	Echocardiogram ejection fraction (abnormal <45%)	Cardiac catheterization	Endomyocardial biopsy	Other
1, Kane	31 F	3/8	Cough, shortness of breath, malaise for 3–5 d, diarrhea for 2 d	Decreased	Normal coronary arteries	Autopsy: lymphocytic infiltration of the myocardium	–
2, La Salle	47 M	3/10	None	15%–20%	None	None	EKG*: new onset atrial fibrillation
3, Kendall	70 M	3/4	Upper respiratory tract infection for 2 wk	20%–25%	None	None	–
4, Kane	45 F	3/10	Fever, shortness of breath, obtundation for 1 d	30%	None	None	–
5, DeKalb	26 F	3/4	Viral bronchitis 1 mo before admission	None	None	None	EKG: ventricular fibrillation arrest
6, Kendall	32 M	1/28	Upper respiratory tract infection and diarrhea for 10 d	20%	Normal coronary arteries	None	–
7, Kane	42 M	03/25	Cough for 2 wk	20%–25%	None	None	EKG: new onset atrial fibrillation
8, Kane	45 F	3/6	Viral illness 3 mo before, increasing palpitations for 3 mo	30%	Normal coronary arteries	None	–
9, Will	33 M	3/19	Upper respiratory tract infection for 5 d, shortness of breath for 2 d	Dilated cardiomyopathy	Normal coronary arteries	Lympohocytic and eosinophilic infiltration	–
10, Fulton	56 M	2/8	Upper respiratory tract infection 1 mo before, fever for 1 d	20%	None	None	–
11, Peoria	38 M	2/9	Upper respiratory tract infection for 1 wk	20%–25%	Normal coronary arteries	None	–
12, Cook	28 M	Unknown	Fevers for 2 wk	20%	None	None	–
13, Cook	60 M	3/20	Fever, cough, shortness of breath for 6 d	Decreased with global hypokinesis	None	None	–
14, Cook†	34 F	2/28	Unknown	Decreased, pericardial effusion	None	None	–
15, DuPage	39 F	2/28	Upper respiratory tract infection symptoms for 1 wk	20%–25%	None	None	EKG: ventricular fibrillation arrest
16, Winnebago	20 M	4/6	Weight loss for 6 wk, vomiting and hemoptysis for 2 wk	None	None	Acute dilated cardiomyopathy	EKG: asystolic arrest

*EKG, electrocardiogram. †Patient 14 had a diagnosis of myopericarditis.

No common exposures could be identified among the patients. None of the patients had recently been vaccinated for smallpox.

Information on acute serologic testing for group B coxsackievirus performed at hospitals was known for 5 patients. Two patients (patients 11 and 14) had elevated antibody titers to group B coxsackievirus. Patient 14 had a convalescent-phase serum specimen collected for group B coxsackievirus antibody testing that had a 2-fold greater titer than the acute-phase sample. Acute serologic testing for echovirus was performed for 2 patients; results were positive for patient 14 and negative for patient 13. Patient 14 also had an elevated acute-phase influenza B antibody titer but a negative convalescent-phase antibody titer. Patient 12 had no change in acute- and convalescent-phase–positive titers for group B coxsackievirus (Table 2).

**Table 2:** Laboratory features of reported myocarditis case-patients, northern Illinois, 2003*

Patient and county of residence	Local test results		California laboratory tests			Outcome
	Group B coxsackie virus serology (reference range <1:8)	Other	Specimens	Date collected	Results	
1, Kane	None	Blood cultures: negative; pericardial fluid culture: negative	Serum, myocardial tissue	3/8, 3/9	Negative	Died
2, La Salle	None	–	Serum	4/2	Negative	Recovered
3, Kendall	None	–	Serum	4/1	Negative	Recovered
4, Kane	None	*Legionella* urinary antigen: negative; sputum, blood, CSF, urine, stool viral cultures: negative	Serum	3/24	Negative	Recovered
5, DeKalb	None	–	Serum	4/2	Negative	AICD, recovered
6, Kendall	None	–	Serum	4/1	Negative	Recovered
7, Kane	Acute: negative	Influenza A and B: negative; blood, throat, urine, stool viral culture: negative	Serum	3/25	Negative	Recovered
8, Kane	None	–	Serum	4/1	Negative	Recovered
9, Will	None	–	Myocardial tissue	3/03	Negative	Recovered
10, Fulton	None	–	None	–	–	Recovered
11, Peoria	Acute: positive 1:80; convalescent: positive 1:80	Mycoplasma IgM serology: negative (reference range < 0.77 U/L); mycoplasma IgG serology: positive 1.47 U/L (reference range < 0.77 U/L); EBV, CMV serology: negative	Serum	4/3	Negative	Recovered
12, Cook	None	Nasopharyngeal, stool culture: negative	None	–	–	Recovered
13, Cook	Acute: negative	Echovirus serology: negative Influenza A and B, RSV rapid tests: negative; *Legionella* urinary antigen: negative	Serum	4/12	Negative	Recovered
14, Cook†	Acute: positive 1:320; convalescent: 1:640	Blood culture: negative; mycoplasma IgM serology: 0.11 negative (reference range < 0.77 U/L); mycoplasma IgG serology: negative 0.07 U/L (reference range < 0.77 U/L); acute echovirus type 11 serology: 1:320 (reference range >1:10); acute influenza A serology: negative (reference range >1:8; acute influenza B serology:positive 1:32; convalescent influenza B serology: negative (reference range <1:8); endotracheal viral culture: negative	None	–	–	Recovered
15, DuPage	Acute: negative	CMV, EBV serology: negative	None	–	–	AICD, recovered
16, Winnebago	None	–	Myocardial tissue	4/7	Negative	Died

*CSF, cerebrospinal fluid; EBV, Epstein-Barr virus; CMV, cytomegalovirus; RSV, respiratory syncytial virus; AICD, automatic interventricular cardiac defibrillator. †Patient 14 had myopericarditis.

IDPH laboratory cultured nasopharyngeal (n = 5), urine (n = 6), stool (n = 6), and myocardial tissue (n = 1) specimens from 9 patients for enterovirus viral isolation. All cultures were negative. Among specimens (serum samples from 11 patients and myocardial tissue from 2 patients) tested for enterovirus and adenovirus by PCR and enzyme immunoassay , all were negative (Table 2).

For the 2 patients with fatal cases, the primary autopsy diagnosis was acute myocarditis. Autopsy tissue specimens from the 1 case-patient submitted to CDC were negative for viral agents (patient 1).

## Conclusions

An outbreak of myocarditis of unknown cause occurred among adults in Kane County (population 400,000) and adjacent areas during winter and early spring 2003. Surveillance for myocarditis cases was initiated throughout Illinois in March and April, although clustering of cases was only evident in and limited to Kane County and surrounding communities. The reporting of myocarditis cases from other counties likely reflected baseline rates of idiopathic myocarditis in those populations that only came to the attention of public health officials through enhanced surveillance.

No common exposures were identified among case-patients. The outbreak occurred within the same period that adverse events of myopericarditis were being reported after smallpox vaccinations among military and healthcare personnel in the United States, including Illinois (*12*); however, no patients in this outbreak had recently been vaccinated against smallpox. Most illnesses were preceded by a prodrome that suggested the outbreak was viral in origin. Substantial illness and death occurred in these reported cases. All reported patients were hospitalized, 2 required AICD devices, and 2 deaths occurred, a reminder of the severe sequelae associated with this illness.

Despite extensive laboratory testing on submitted specimens, no specific agent was identified. Cross-reactivity of group B coxsackievirus serology with several agents was apparent from initial laboratory tests performed at the hospitals. These results were insufficient to support a specific cause of illness. Tissue specimens from only 3 of the 16 patients were available for testing, which was a major laboratory limitation in the investigation, particularly for detecting viral nucleic acid by PCR assays. The inability to implicate a responsible agent is a common outcome of myocarditis outbreak investigations (*1**,**13*).

A better understanding of myocarditis through enhanced diagnostic and therapeutic strategies, increased awareness of possible clusters of illness, and rapid reporting of clusters to public health departments will help improve prevention of future outbreaks. Recent biopsy-based studies suggest that a proportion of life-threatening myocarditis or idiopathic cardiomyopathy in otherwise healthy adults may arise from enteroviral and cytomegalovirus infections (*14**,**15*). Research is needed to assess the effect of potential antiviral treatment on illness and death in this patient population. In addition to encouraging appropriate viral testing of acute- and convalescent-phase serologic specimens, further study is required to examine the usefulness of endomyocardial tissue collection for advanced molecular analyses in patients with unexplained cardiomyopathy.

## Appendix

Selected Codes for Myocarditis and Pericarditis from the International Classification of Diseases, 9th Revision, Clinical Modification (ICD-9-CM).

Myocarditis: 422.90, 422.92, 422.93, 422.99

Other primary cardiomyopathies: 42.4
